# Multiplex CRISPR/Cas9-mediated genome editing of the *FAD2* gene in rice: a model genome editing system for oil palm

**DOI:** 10.1186/s43141-021-00185-4

**Published:** 2021-06-11

**Authors:** Bohari Bahariah, Mat Yunus Abdul Masani, Omar Abd Rasid, Ghulam Kadir Ahmad Parveez

**Affiliations:** Advanced Biotechnology and Breeding Centre (ABBC) Division, Malaysian Palm Oil Board (MPOB), 6, Persiaran Institusi, Bandar Baru Bangi, 43000 Kajang, Selangor Malaysia

**Keywords:** Genome editing, Multiplex CRISPR/Cas9, FAD2, High oleic acid, Rice, Model monocot

## Abstract

**Background:**

Genome editing employing the CRISPR/Cas9 system has been widely used and has become a promising tool for plant gene functional studies and crop improvement. However, most of the applied CRISPR/Cas9 systems targeting one locus using a sgRNA resulted in low genome editing efficiency.

**Results:**

Here, we demonstrate the modification of the *FAD2* gene in rice using a multiplex sgRNA-CRISPR/Cas9 genome editing system. To test the system’s efficiency for targeting multiple loci in rice, we designed two sgRNAs based on *FAD2* gene sequence of the *Oryza sativa Japonica* rice. We then inserted the validated sgRNAs into a CRISPR/Cas9 basic vector to construct pYLCRISPRCas9PUbi-H:OsFAD2. The vector was then transformed into protoplast cells isolated from rice leaf tissue via PEG-mediated transfection, and rice calli using biolistic transformation. Direct DNA sequencing of PCR products revealed mutations consisting of deletions of the DNA region between the two target sgRNAs.

**Conclusion:**

The results suggested that the application of the multiplex sgRNA-CRISPR/Cas9 genome editing system may be useful for crop improvement in monocot species that are recalcitrant to genetic modification, such as oil palm.

**Supplementary Information:**

The online version contains supplementary material available at 10.1186/s43141-021-00185-4.

## Background

Genetic engineering of crops by the clustered regularly interspaced short palindromic repeats (CRISPR)/CRISPR-associated protein 9 (Cas9) genome editing is the most important breakthrough technology of the decade. CRISPR/Cas9 system adapted from immune system type II, which has been found in bacteria *Streptococcus pyogenes.* This CRISPR/Cas9 system can efficiently induce targeted mutations based on base-pairing of the engineered single-guide RNAs (sgRNAs) to the target DNA sites [1]. The process relies on the use of sequence-specific nucleases that can be induced to recognize specific DNA sequences and subsequently generate double-strand breaks (DSBs). The DSBs are then repaired by two endogenous mechanisms by either non-homologous end joining, which lead to small insertion or deletion of nucleotides, thereby causing gene knockouts [2], or by homologous recombination, which can cause gene replacements and insertions [3] that lead to the loss of gene function. The CRISPR/Cas9 system is a powerful and efficient tool for targeted mutagenesis with the potential for editing multiple genomic loci and generating a range of gene mutations [4]. There have been several recent reports of CRISPR/Cas9-mediated targeted mutagenesis in a variety of monocots, including rice (*Oryza sativa*), wheat (*Triticum* sp.), barley (*Hordeum vulgare*), and maize (*Zea mays*) [5–10].

CRISPR/Cas9 has also been widely used for functional study of rice genes which was first reported in 2013 with the rice *phytoene**desaturase* as the target gene [11]. This approach has also been used to develop rice plants with high oleic acid content [12]. High oleic oil is high in monounsaturated fats and low in polyunsaturated fats, characterized at 70% or more oleic acid content. Increasing the monounsaturated fatty acids would enhance the physical properties that can be used in products that need to be shelf-stable with a longer shelf life, a higher heat tolerance, and more nutritional aspects [12]. Besides, a large amount of monounsaturated fat in high oleic oil has been proven to help prevent lifestyle diseases and increase health benefits [13]. Modification of the fatty acid synthesis pathway via suppression of the oleoyl-CoA desaturase (FAD2) will result in the reduction of linoleic acid formation and increase in oleic acid accumulation. In rice genome, four *FAD2* genes, designated as *OsFAD2-1*, *OsFAD2-2*, *OsFAD2-3* and *OsFAD2-4* were identified [13]. Among them, the *OsFAD2-1* gene is the most highly expressed *FAD2* gene in rice seeds. Thus, the knockout of *OsFAD2-1* by CRISPR/Cas9 could lead to a high oleic acid content in rice. Previously, a CRISPR/Cas9 vector harbouring single sgRNA targeting *OsFAD2-1* gene has been constructed and transformed into rice calli through *Agrobacterium*-mediated transformation [12]. Rice transgenic mutants were successfully generated, and the impact on fatty acid profiles was evaluated. However, the significant increase of oleic content could only be observed in T2 rice homozygous mutant lines. The oleic acid content in T2 rice homozygous for the OsFAD2-1 mutant allele increased to more than double, from 32 to 80% of the total fatty acid composition, and interestingly, no linoleic acid was detected. Meanwhile, in T2 heterozygous mutant lines, the oleic acid content only increased slightly at about 10% with a concomitant 10% reduction of linoleic acid content as compared with wild-type rice. The study indicated that using a single sgRNA to knockout the *OsFAD2-1* gene is not very efficient as homozygous mutant lines could only be obtained in T2 generation lines.

Therefore, in this study, multiple sgRNAs were used to test the plant genome editing system to have a higher homozygous and biallelic mutation rate in T0 plants to get higher oleic acid–producing plants. The findings of previous studies [14–16] demonstrated that targeting two or more sgRNAs for one gene were highly efficient and showed high homozygous and biallelic mutations rate in the T0 plants. Studies from [15, 16] resulted in high mutation rates of 82.2%, which subsequently generated 28.1% of the homozygous and 56.7% biallelic mutant lines. The gene mutations were stable to the next generation (T1) following the classic Mendelian law. However, several factors could affect the specificity and efficiency of sgRNAs. Thus, it is recommended that multiple sgRNAs be selected to target a gene of interest.

This study aims to get high oleic acid–producing plants by manipulating the *OsFAD2-1* gene in rice but using a multiplex sgRNA-CRISPR/Cas9 genome editing system. We used a robust CRISPR/Cas9 vector system utilizing a plant codon-optimized *Cas9* gene as a convenience system [17] to manipulate the *OsFAD2-1* gene in rice by employing multiple sgRNAs for high-efficiency genome editing capabilities. Using the vector system, we have successfully constructed a pYLCRISPR/Cas9 plasmid with two different CRISPR/Cas9 target sites within the rice *OsFAD2-1* gene, namely OsFAD2-T1 and OsFAD2-T2. We then delivered the plasmid DNA into protoplasts by a PEG-mediated method and at the same time into rice calli by biolistic transformation. We found nearly a 300-bp deletion from both transformation experiments. As rice is a model plant for monocots, the vector system and information obtained from this study can also be used for crops with more complicated genomes, such as oil palm, for genome research of gene function and trait improvement.

## Methods

### Design of sgRNA

The target sequences of sgRNA were selected and designed using the online tool CRISPR-GE (http://skl.scau.edu.cn/) [17]. The sgRNA sequences were designed based on the genomic sequences of *OsFAD2-1* fatty acid desaturase DES2 [*O. sativa* Japonica Group (Japanese rice)] located on chromosome 2 (NCBI link: https://www.ncbi.nlm.nih.gov/gene/4330523). The CRISPR-GE can detect Protospacer Adjacent Motif (PAM) sequences and lists possible sgRNA sequences within a specific DNA region. A unique genomic potential target of 20 nucleotide DNA sequence present immediately upstream of a PAM site at 3′ (5′-20nt-NGG-3′) was selected by comparing to the rest of the genome. Potential target sequences with dozens of bases forward and reverse were examined for sequence similarity using Blast at NCBI (https://www.ncbi.nlm.nih.gov) against the rice (*O. sativa japonica*) genome with somewhat similar sequences option. This was carried out to confirm their targeting specificity in the genomes. Two candidates of target sgRNA sequences were selected based on their GC content (greater than 40% for high editing efficiency), and the absence of four or more consecutive “T” nucleotides in the target sequence as the sequence would be recognized as a transcriptional termination signal by RNA polymerase III [16]. The secondary structures of target-sgRNA sequences (20-bp target) linked to nucleotides of the sgRNA scaffold (GTTTTAGAGCTAGAAATAGCAAGTTAAAATAAGGCTAGTCCGTTATCAACTTGAAAAAGTGGCACCGAGTCGGTGCTTTTTTT) were analysed with the RNA Folding Form programme (http://mfold.rna.albany.edu/?q=mfold/RNA-Folding-Form2.3). The software was used to ensure good base pairings between the target sequence and the sgRNA sequence, greatly affecting the editing efficiency [18].

### In vitro validation of sgRNA

An in vitro assay to determine the most effective sgRNAs was carried out prior to delivery into the cells. The efficiencies of sgRNAs were evaluated by using the Guide-it™ sgRNA In Vitro Transcription and Screening Systems Kit (Takara Bio USA, Inc). Briefly, PCR was performed with a 56- to 58-nt customized oligonucleotide forward PCR primer (Supplementary Table 1) that contained the T7 promoter (TAATACGACTCACTATA), the 20-nt sgRNA DNA targeting sequence, and the 15-nt scaffold template annealing (GTTTAAGAGCTATGC). PCR amplification was performed using the following condition: 33 cycles of 98 °C for 10 s and then 68 °C for 10 s. PCR product of ~ 130 bp was purified and used as a template for a T7 RNA polymerase–mediated transcription reaction using Guide-it T7 Polymerase Mix (Takara Bio USA, Inc). The transcribed sgRNAs were then purified, and the concentration was determined using a NanoDrop 2000 spectrophotometer (Thermo Fisher). The transcribed sgRNA, Cas9 protein, and targeted DNA fragment were mixed for in vitro cleavage assay according to the manufacturer’s protocol (Takara Bio USA, Inc). The resulted cleavage assay products were electrophoresed on a 2% agarose gel to determine the cleavage potential at the DNA target site. Trans2K® Plus II DNA Marker (Cat. No. BM111-01, TransGen Biotech, China) and 100 bp plus DNA Ladder (Cat. No. BM321-01, TransGen Biotech, China) were used as molecular weight standards for agarose gel electrophoresis.

### Construction of multiplex sgRNA CRISPR/Cas9 vector

In order to construct the CRISPR/Cas9 vector with multiplex sgRNA, two intermediate expression cassettes containing the sgRNA sequence were constructed according to the method described by Ma et al. [17]. Briefly, the 20-nt target sequences were synthesized using overlapping PCR. In the first round of PCR, the 20-nt target sequences were ligated into its corresponding sgRNA expression cassettes (pYLsgRNA-OsU6a and pYLsgRNA-OsU6b, obtained from Dr. Yao-Guang Liu’s laboratory, South China Agricultural University, China). This was followed by a second PCR to combine the two fragments of sgRNA expression cassettes flanked with two *Bsa*I restriction sites. Amplified PCR products containing sgRNA expression cassettes were then cloned into *Bsa*I site of pYLCRISPR/Cas9PubiH (obtained from Dr. Yao-Guang Liu’s laboratory, South China Agricultural University, China), via the Gibson assembly cloning method [19]. Positive clones were confirmed by PCR, *Asc*I digestion, and DNA sequencing. All the primer sequences used in this study are listed in Supplementary Table 1.

### Plant materials

Mature seeds of rice (*O. sativa* L.) cultivar Nipponbare were dehulled and sterilized with 70% ethanol for 1 min (Supplementary Fig. 1). The seeds were further sterilized with 2.5% sodium hypochlorite for 20 min and washed at least five times with sterile water. The sterile seeds were then incubated on 1/2 MS medium with a photoperiod of 12-h light and 12-h dark at 26 °C for 10–14 days. Two-week-old plants were used for protoplast isolation. Meanwhile, rice calli (*O. sativa* L.) cultivar Nipponbare were cultured on a callus induction medium for 1 month. Embryogenic calli obtained were used as target material for bombardment.

### Protoplast isolation and PEG-mediated protoplast transfection

**Fig. 1:**
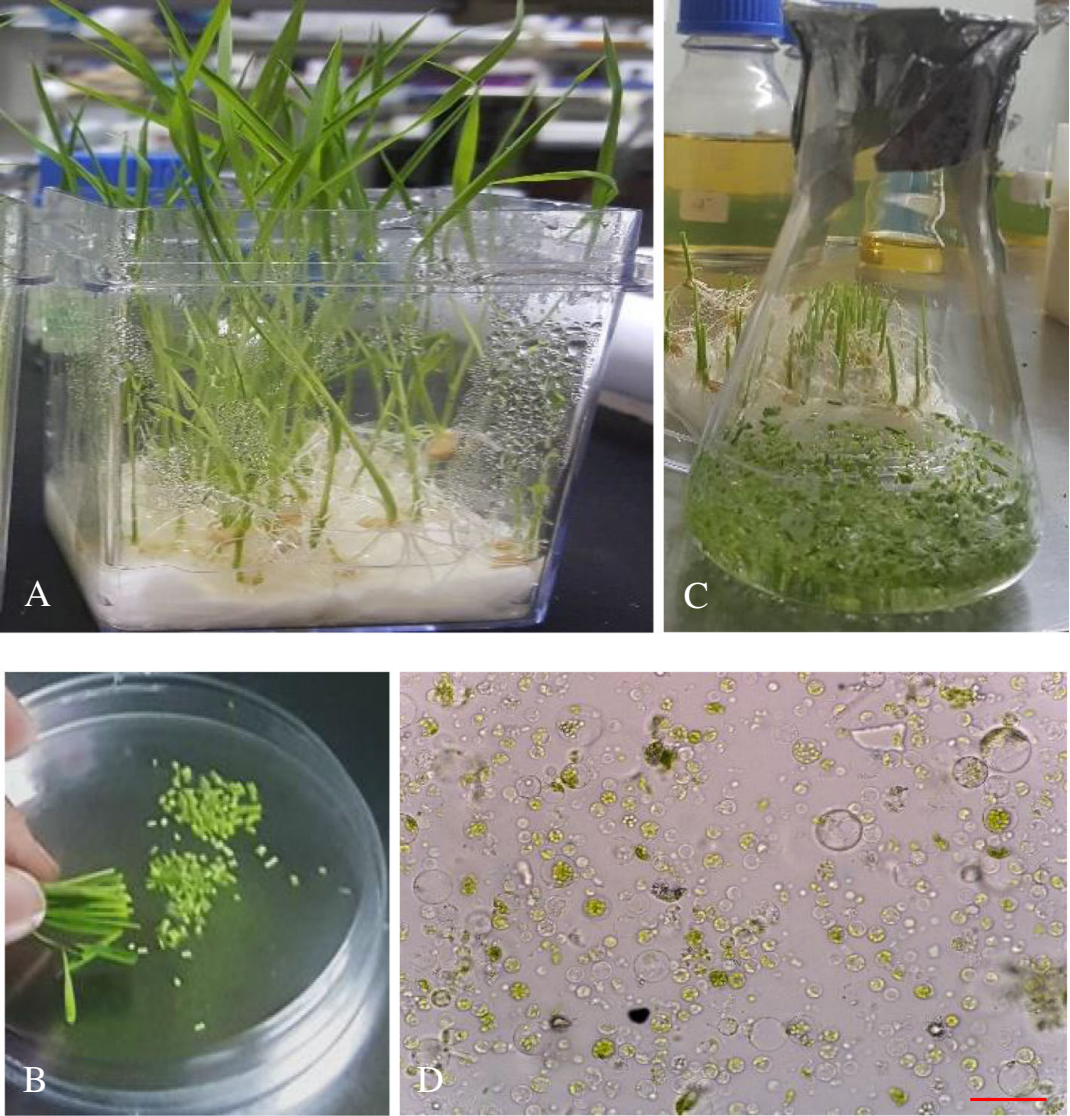
Fourteen-day-old rice seedlings used for protoplasts isolation (**A**). Stem and sheath of the seedlings were cut into approximately 0.5-mm strips (**B**). The strips were treated with 0.6 M mannitol (**C**) followed by enzymatic digestion. Protoplast image under the microscope (**D**). Bar in (**D**) is 100 μm

Rice protoplasts were isolated following the methods described by Zhang et al. [20] and Yoo et al. [21] with some modifications. The green tissues from the stem and sheath of two-week-old rice seedlings were used for protoplast isolation (Fig. 1A). About 30 seedlings were cut into 0.5-mm strips with a sterile razor blade (Fig. 1B). The leaf strips were immediately immersed into 0.6 M mannitol for 10 min in the dark (Fig. 1C). The strips were then incubated in an enzyme solution which consisted of 1.5% (w/v) cellulase R10, 0.75% (w/v) macerozyme R-10, 0.6 M mannitol, 10 mM MES at pH 5.7, 10 mM CaCl_2_, and 0.1% (w/v) BSA for 6 h in the dark with gentle shaking (60–80 rpm). An equal volume of W5 solution (154 mM NaCl, 125 mM CaCl_2_, 5 mM KCl, and 2 mM MES at pH 5.7) was added, and the digestion mixture was gently shaken for 1 h. The released protoplasts were filtered through a 40-μm nylon mesh into a round bottom tube. The protoplasts were collected by centrifugation at 1000 rpm for 5 min with a swing bucket. After one washing step with W5 solution, the pelleted protoplasts were resuspended in 500 ml MMG solution (0.4 M mannitol, 15 mM MgCl_2_, and 4 mM MES at pH 5.7). The protoplasts were transferred to a slide, and the viability was checked by microscopy (Olympus, Tokyo, Japan) (Fig. 1D). For each transfection, 2 μg of total plasmid DNA were mixed with 100 μl protoplasts. Then, 100 μl freshly prepared PEG solution [40% (w/v) PEG 4000, 0.2 M mannitol, and 0.1 M CaCl_2_] was added, and the mixture was gently mixed and incubated at room temperature for 20 min in the dark. After incubation, 1 ml W5 solution was added. The resulting solution was mixed well by gently inverting the tube, and the protoplasts were pelleted by centrifugation at 1000 rpm for 5 min. The protoplasts were resuspended gently in 1 ml W5 solution. Finally, the transfected protoplasts were incubated in the dark at room temperature for 24–48 h.

### Genomic DNA extraction and mutation validation in transfected protoplasts

Genomic DNA was extracted from pooled protoplasts with four replications. The protoplasts cells were lysed by adding an equal volume of chloroform and allowing the phases to separate by centrifugation at 13,000 rpm for 15 min. The clear upper layer (aqueous phase) was transferred into a new tube, and an equal volume of isopropanol was added. The mixture was mixed gently and incubated at – 80 °C. After 1 h, the DNA was collected by centrifugation at 13,000 rpm for 15 min. The supernatant was discarded, and the pellet was washed with 1 ml 100% ethanol. The ethanol was discarded, and the pellet was air-dried and dissolved in 50 μl sterile water. Two pairs of primers were designed to amplify the targeted genomic region. The 25 μl PCR reaction contained approximately 100 ng template DNA, 1 μl (10 μM) specific forward and reverse primers, and 12.5 μl of 2× T5 Super PCR Mix (TsingKe Co. Ltd., Beijing, China). The primers used are listed in Supplementary Table 1. The purified PCR products were sequenced. Targeted gene mutations were detected by aligning the sequencing chromatograms of these PCR products with the wild-type controls using the SNAPGENE Tools software.

### Biolistic transformation of rice calli

Plasmid DNA for the bombardment of rice calli was isolated using a QIAGEN Hi-Speed Plasmid Midi Kit (Qiagen, USA) according to the manufacturer’s instructions. A total of 5 μg/μl of plasmid DNA was mixed with 0.6-μm gold particles. About 30 to 40 embryogenic calli with 3- to 4-mm sizes were arranged at the centre of the plate containing a callus initiation medium. The calli were bombarded at 1100 psi, with a target distance of 60 mm, using the PDS-1000/He™ device according to manufacturer instruction (Bio-Rad, USA). The bombarded calli were incubated in the dark at 26 °C for callus initiation.

### Genomic DNA extraction and mutation detection in bombarded calli

Total genomic DNA of bombarded rice calli (1 to 5 mg) was isolated 2 months after transformation according to CTAB protocol [22]. The extracted genomic DNA was then used as a template to amplify the desired fragments in each of the target genes using primers flanking the target site (Supplementary Table 1). PCR amplification reaction was performed using KOD-FX DNA Polymerase (TOYOBO-KOD FX) in a final volume of 20 μl. PCR was carried out using an Eppendorf AG 22331 Hamburg Mastercycler (Eppendorf) for 36 cycles consisting of denaturation at 95 °C for 30 s, annealing at 60 °C for 30 s, and extension at 72 °C for 30 s. The amplified products were cloned into the pEASY-Blunt cloning vector (TransGen Biotech, China). The resulting ligation product was subsequently transformed into *Escherichia coli Trans*1-T1 competent cell and spread onto LB plates supplemented with kanamycin. Putative colonies were screened by a colony PCR, and the insert DNA was sequenced. The mutations were analysed by DSDecodeM (http://skl.scau.edu.cn/dsdecode/), an online analysis tool [17] for decoding multiple Sanger sequencing chromatograms derived from PCR amplicons. The degenerate sequence decodes superimposed sequencing peaks containing various types of mutations such as biallelic, heterozygous, and homozygous mutations. The sequences (ab1 format) with the sequence information and the reference sequence (WT) were uploaded and decoded. The results were to verify the deletions, insertions, substitutions, and type of mutations in the target region of sgRNA.

## Results

### sgRNA design and validation of cleavage efficiency via in vitro Cas9 cleavage assay

**Fig. 2:**
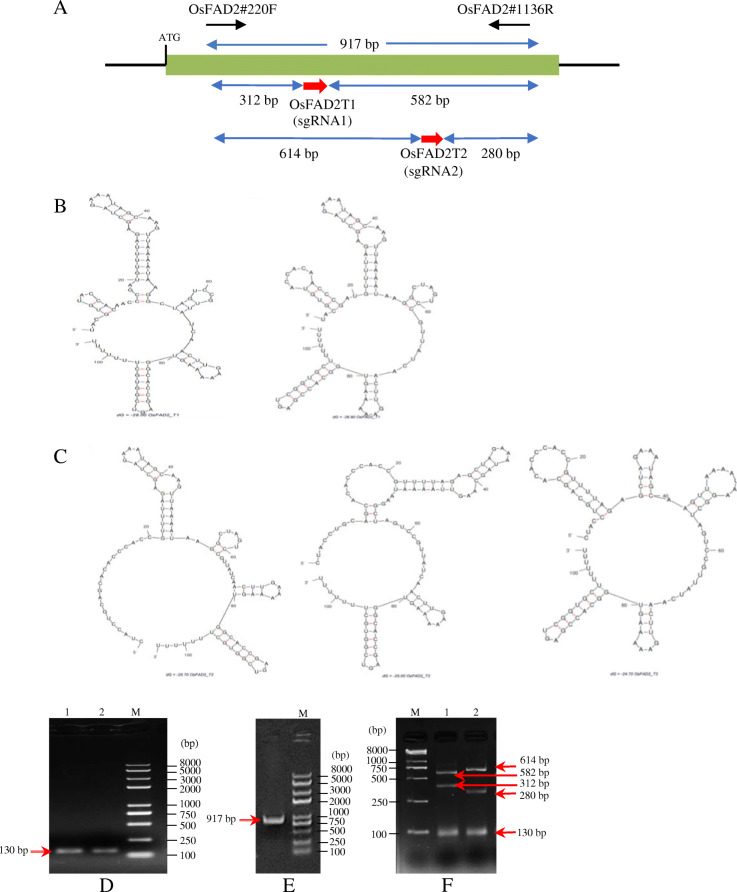
**A** Schematic of *OsFAD2-1* gene loci. Predicted secondary structures for (**B**) sgRNA1 and (**C**) sgRNA2. PCR amplification and validation of sgRNAs via in vitro Cas9 cleavage assay; **D** PCR product from amplification of sgRNAs template at ~ 130 bp (lane 1: sgRNA1, lane 2: sgRNA2) for in vitro transcription, **E** PCR amplification of rice target DNA region (OsFAD2T1T2) at ~ 917 bp, and (**F**) analysis of cleavage products for sgRNA1 at 312 bp and 582 bp (lane 1) and sgRNA2 at 614 bp and 280 bp (lane 2). Band size at ~ 130 bp in (**F**) at lanes 1 and 2 are the residue of sgRNA template. Red arrows indicate the band size at the expected molecular weight. Lane M is Trans2K® Plus II DNA Marker (TransGen Biotech, China)

In order to disrupt the *FAD2* gene, two target sites on its coding sequence (CDS) region located on the exon two were selected, and the primer pairs (OsFAD2#220F and OsFAD2#1136R) flanking the two sgRNAs were designed (Fig. 2A). The two designed sgRNAs, namely OsFAD2-T1 (5′ TACGTGTACCACAACCCGATCGG 3′) and OsFAD2-T2 (5′ CTACCTGCAGCACACCCACCCGG 3′), comprised of 20 nucleotide sequences and immediately followed by nucleotides of the PAM sequences (5′-NGG). The selected sgRNAs had 50% and 65% GC content, respectively. For the selection of efficient sgRNAs that affect the editing ability of the CRISPR/Cas9 system, sgRNAs with a GC content between 50 and 75% are desirable [16]. This range of GC contents in the target sequences could result in relatively higher editing efficiencies [17].

Based on the secondary structure of target sequences, the predicted number of unpaired or mismatched nucleotides in the targeted regions of the RNA sequence for both sgRNAs, OsFAD2-T1 and OsFAD2-T2, was shown to contain no more than six mismatches to the target sequences (Fig. 2). The two structures were predicted for sgRNA1 (OsFAD2-T1) containing 4 and 5 mismatched nucleotides (Fig. 2B) and three structures containing 0, 3, and 6 mismatches for sgRNA2 (OsFAD2-T2) (Fig. 2C). The predicted secondary structure of the OsFAD2-T1 and OsFAD2-T2 seed regions may potentially improve the cleavage activity of Cas9 and sgRNA performance. It was suggested that the secondary structure of sgRNA is an important parameter and critical factor to achieve high on-target efficiency of the CRISPR/Cas9 system [23].

A fast and sensitive in vitro Cas9 cleavage assay for sgRNA validation was also done before applying the sgRNAs into plant cells. The main advantage of in vitro Cas9 cleavage assay is that the evaluation of the target specificity in vitro is independent of the genome or species used. As shown in Fig. 2D, 130-bp PCR templates for in vitro transcription of sgRNAs were successfully amplified. The PCR products containing the T7 promoter sequence fused with the variable 20-nt sgRNA target sequence (OsFAD2-T1 and OsFAD2-T2) followed by constant region sequences. The PCR products were then transcribed in vitro with T7 polymerase–synthesized sgRNAs. A 917-bp PCR amplicon containing a target region was amplified using primers, OsFAD2#220F and OsFAD2#1136R, from wild-type *Japonica* rice cultivar Nipponbare genomic DNA (Fig. 2E). According to the protocol, the targeted DNA fragment, sgRNAs, and recombinant Cas9 nuclease were combined for in vitro cleavage reaction. The efficiency of two sgRNAs to cleave the target DNA fragment was verified when no band at a size of 917 bp was observed (Fig. 2F). The 917-bp DNA fragment was completely cleaved by sgRNA1/Cas9 ribonucleoprotein complexes to form two DNA fragments with sizes of 312 bp and 582 bp. Meanwhile, two fragments of sizes 614 bp and 280 bp were observed from sgRNA2/Cas9 ribonucleoprotein complexes, suggesting both sgRNAs were efficient to introduce mutation or deletion within the target region.

### CRISPR/Cas9 expression vector construction

**Fig. 3:**
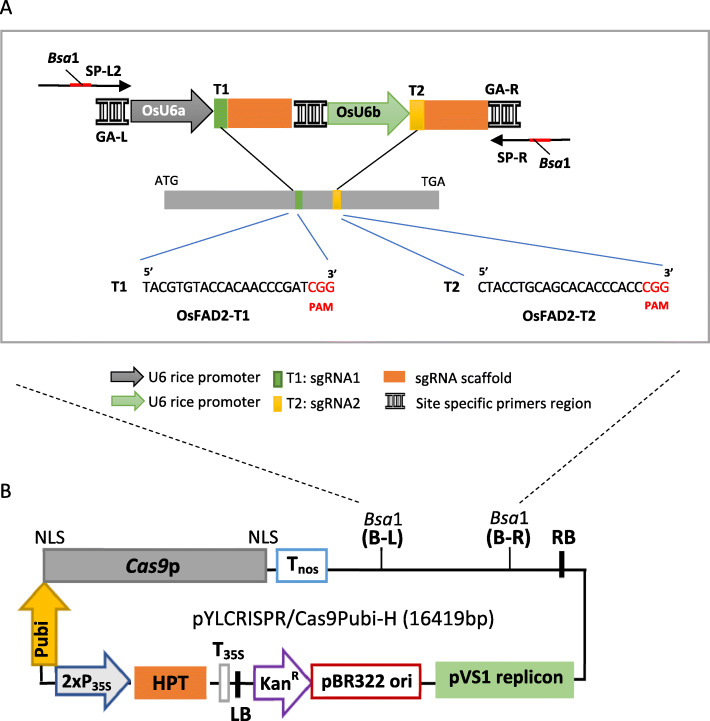
Strategy for the construction of pYLCRISPR/Cas9 targeting OsFAD2-1 in rice. **A** Schematic illustration of target sites for two sgRNA expression cassettes driven by U6 promoters from rice; *OsU6a* promoter for OsFAD2-T1 and *OsU6b* promoter for OsFAD2-T2, the 20-nt sequences in green square boxes and orange square boxes indicate the PAM motif of each sgRNA, the *Bsa*I restriction enzyme sites for the entry of multiple sgRNA expression cassettes, SP-L2 and SP-R are sequencing vector-specific primers, GA-L and GA-R are GA site-specific primers for the Gibson assembly of sgRNA expression cassettes; **B** pYLCRISPR/Cas9PubiH basic vector has two *Bsa*I sites for insertion of two sgRNA expression cassettes [17] (LB: left border of T-DNA, T35S: cauliflower mosaic virus *35S* terminator, *HPT*: gene for hygromycin phosphotransferase, 2xP*35S*: Double cauliflower mosaic virus *35S* promoter, P*ubi*: maize ubiquitin *Ubi1* promoter, NLS: Nuclear localization sequence. *Cas9*p: CRISPR associated protein 9, Nos*: nopaline synthase* gene terminator, RB: right border of T-DNA, pVS1 replicon: pVS1 for replication, pBR322: pBR322 vector, Kan^R^: Kanamycin resistance gene)

In order to test the ability and efficiency of multiple sgRNA for genome editing in rice, two sgRNAs expression cassettes targeting two adjacent sites of the *OsFAD2-1* gene were assembled in a single vector. These two sgRNA sequences are driven by their own individual promoter, which has been constructed using a robust CRISPR/Cas9 vector system developed by Ma et al. [17]. As illustrated in Fig. 3, two multiple sgRNA expression cassettes were assembled into the *Bsa*I-linearized pYLCRISPR/Cas9Pubi-H binary vector by the Gibson assembly method to create pLYCRISPRCas9PUbi-H:OsFAD2. The *E. coli* colonies carrying the pLYCRISPRCas9PUbi-H:OsFAD2 plasmid were identified by colony PCR and restriction enzyme digestion with *Asc*1 (Supplementary Fig. 2). In addition to these molecular detection methods, validation by Sanger sequencing of the positive colonies using the primer pair SP-L1 and SP-R was also performed. Nucleotide blast from the NCBI webpage used for sequence comparison confirmed that the PCR product contained the vector sequences (supplementary Fig. 3).

The pYLCRISPRCas9PUbi-H:OsFAD2 vector utilizing a plant codon-optimized*Cas9* gene (*Cas9p*) was used to induce and improve the editing efficiency in monocots using rice as a testing platform [17]. In addition, the vector also contains an *RNA polymerase III* (*Pol III*) promoter, *OsU6*, a monocot-derived promoter, as a strategy to increase the transcription of the sgRNA in monocot plants. It was reported that sgRNA regulated by the *OsU6* promoter produced more transcript levels than the *OsU3* promoter [24]. Due to this, pYLCRISPRCas9PUbi-H:OsFAD2 has two types of *OsU6* promoters, namely *OsU6a* and *OsU6b*. The *OsU6a* is derived from an *Indica* rice cultivar 93-11, while *OsU6b* originated from a *Japonica* rice cultivar Nipponbare [17]. Both promoters are used as a strategy to control multiple sgRNA. The pYLCRISPRCas9PUbi-H:OsFAD2 binary vector also contains the *hygromycin phosphotransferase* (*HPT*) gene driven by the maize ubiquitin promoter for the selection of transgenic plants.

### Detection of multiplex genome editing in transfected protoplasts and bombarded calli

PEG-mediated transfection of employing rice protoplasts isolated from young green tissue is an alternative testing strategy before performing a stable transformation. This is an easy and fast procedure to evaluate the CRISPR/Cas9 genome editing system. Several studies have shown the use of protoplasts as a useful and convenient protocol to determine the effectiveness of designed sgRNAs and confirm the sgRNA/Cas9 editing efficiency [11, 15, 25–31].

**Fig. 4:**
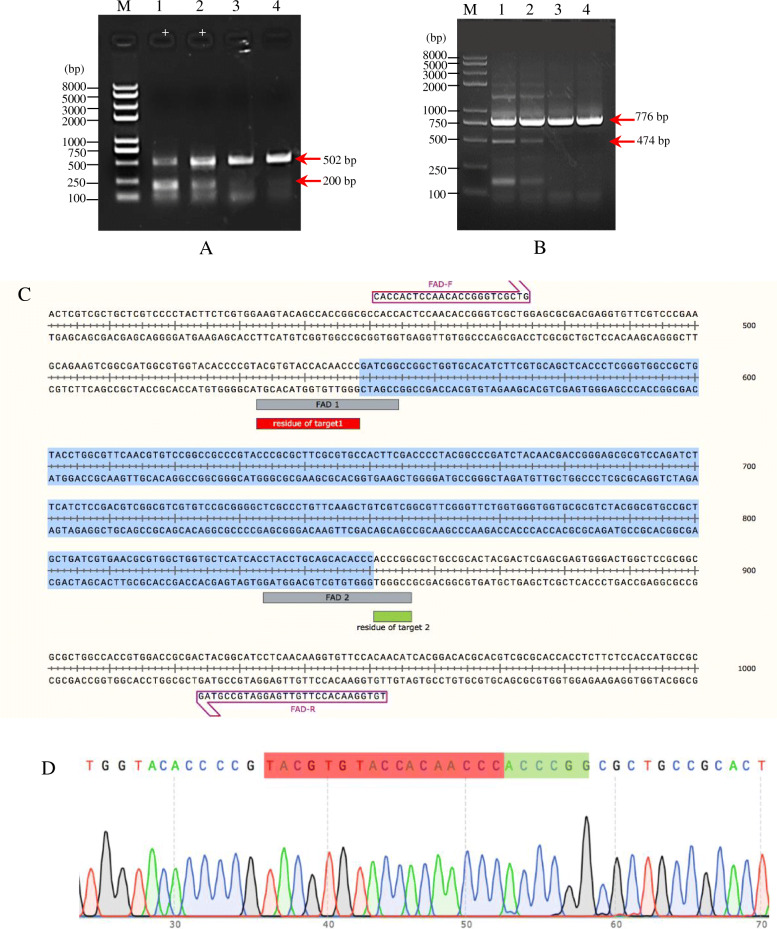
PCR-amplified DNA from protoplasts transfected with pYLCRISPRCas9Pubi-H:OsFAD2. **A** DNA fragments of 502 bp and 200 bp were amplified using FAD2-F and FAD2-R primers. **B** DNA fragments of 776 bp and 474 bp were amplified from second set PCR using FAD2-F2 and FAD2-R primers. Lane M is Trans2K® Plus II DNA Marker (TransGen Biotech, China), and lanes marked with “+” are positive samples. **C** PCR products at 474 bp were directly sequenced and analysed using SNAPGENE revealed mutations consisting of deletions in the DNA region between sgRNA1and sgRNA2. The blue background part is the deleted sequences; the first line (sequence of the wild type target gene) and target site in grey boxes were highlighted. **D** Sequencing chromatogram, the red box is the residue of target 1 (OsFAD2-T1), and the green box is the residue of target 2 (OsFAD2-T2)

The transfected protoplasts with four replications were isolated for genomic DNA isolation. The genomic DNA was used as a PCR template for the target gene amplification (OsFAD2-T1 and OsFAD2-T2). Two sets of primers were used for their specificities and sensitivities in PCR for *OsFAD2-1* gene detection. The first pair, OsFAD2-F and OsFAD2-R primers generate two fragments of 502 bp and 200 bp (Fig. 4A). Meanwhile, the second set of primers (OsFAD2-R and OsFAD2-F2) generated amplicons with sizes of 776 bp and 474 bp (Fig. 4B). However, the amplification with the second set of primers seems to be more suitable for high-quality sequencing analysis of about 400 to 700 bp [32]. Double bands obtained could be due to the indel mutation generated. PCR products obtained using OsFAD2-R and OsFAD2-F2 primers flanking the targeted region at 474 bp were directly sequenced.

The sequences were aligned with the reference sequence of the OsFAD2-1 and compared to the rice genome. Two (events 1 and 2) out of 4 events tested showed deletions of 302 bp, suggesting that Cas9 cuts 3 bp upstream of the PAM for sgRNA1 and 3 bp upstream of the PAM for sgRNA2 (Fig. 4C). The sequencing chromatogram consisting of the residues of sgRNA1 and sgRNA2 after the deletion occurred is shown in Fig. 4D. This observation demonstrated that the non-homologous end-joining repair mechanism is used to repair the DSBs created by the Cas9. Previous studies in monocot also reported that using multiple sgRNA target regions resulted in targeted deletions of 10-bp to over 200-kb nucleotides between two target sites in maize [33].

On the other hand, the biolistic-mediated method was used to generate transgenic rice lines transformed with the plasmid DNA, pYLCRISPRCas9PUbi-H:OsFAD2. Two weeks after the bombardment, the bombarded rice calli were cultured on hygromycin selection media for 2 months. Hygromycin-resistant calli produced were then subjected to PCR and DNA sequencing to detect mutations (Supplementary Fig. 4). Twenty-four resistant calli (T0) were randomly selected for PCR analysis. Of these, five samples showed amplified PCR products at 776 bp and 474 bp were directly sequenced. Sequence analysis of PCR amplicon confirmed the mutant status. The deletion was between sgRNA1 and sgRNA2 target sites, precisely occurred as expected (Supplementary Fig. 4B and 4C). The sequencing result of amplicon derived from transformants was also decoded using DSDecodeM [32] (Supplementary Fig. 5). Two different homozygous mutant alleles for two amplified PCR products were detected. A nucleotide (1 bp) insertion for amplicon 776 bp and a large deletion of 302 nucleotides for smaller amplicon 474 bp in both alleles were identified in the T0 rice. Even though there was no result from fatty acid analysis from this study, the detection of homozygous mutations through molecular analyses probably could result in the knockout of the *OsFAD2-1* gene, subsequently increasing the oleic acid content in rice seeds. This result is consistent with a previous study that reported homozygous mutants in the rice T0 [34].

## Discussion

The development and improvement of the CRISPR/Cas9 system are being established as the preferred method for gene manipulation in the crop. Its efficiency has been demonstrated in model plants such as *Arabidopsis*, rice, and tobacco [35–37]. Rice is a diploid and monocot with a small genome size (430 Mb) plant that has been one of the choices for CRISPR/Cas9 application model systems. Furthermore, DNA transformation of rice is efficient and relatively easy to produce transgenic plants, making it a useful plant for studying biology. Therefore, in this study, we used rice as a model system in the attempt to develop a genome editing system for oil palm. The genomic sequences of the rice (*O. sativa*) genome were found to have significant similarities to the oil palm genomic sequences [38, 39], which can be exploited in oil palm research. This also provides an opportunity to further investigate molecular genetics linked to other oil palm traits by using rice as the reference genome.

Thus, in parallel to this research, efforts are focused on achieving the ultimate goal of the oil palm genetic engineering programme to increase the production of oleic acid content in palm oil by manipulating the *FAD2* gene of the fatty acid biosynthesis pathway [40–42]. In plant, reducing or knocking out the expression of the *FAD2* gene will reduce the percentage of linoleic acid and subsequently will increase the level of oleic acid. Most plants have several *FAD2* genes; rice has four *FAD2* genes and oil palm has two *FAD2* genes, namely *EgFAD2-1* and *EgFAD2-2*. In this work, a robust CRISPR/Cas9 system for multiplexed plant genome editing originally developed by Ma et al. [17] has been conveniently utilized to target the *FAD2* gene of the rice genome. This system is efficient for linking multiple sgRNA cassettes in a single reaction. More than one sgRNA was recommended to target multiple sites in a single gene to improve editing rates, especially in crops with low transformation efficiencies such as oil palm. Currently, the oil palm transformation efficiency is 0.7% for *Agrobacterium*-mediated plant transformation [43], 1.5% for particle bombardment method [44], and 14% for protoplast DNA microinjection [45].

To further improve the transformation efficiency, the potential of the multiplex CRISPR/Cas9 technique was explored by designing two sgRNAs, OsFAD2-T1 and OsFAD2-T2, to interrupt the rice *OsFAD2-1* gene function. For efficient sgRNA target selection, highly specific nucleotide sequences to the genome with GC content 50% and 65% were preferred to reduce off-target effects and produce high editing efficiency. In addition, a plant codon-optimized *Cas9* gene (Cas9p) with higher GC contents (62.5%) in the 5′ region (400 bp) has been used to direct the sgRNAs efficiently. The multiplex CRISPR/Cas9 vector cassettes were created with a construct composed of *Cas9* expressed under the control of *Pol II Ubi1* promoter. Meanwhile, the *Pol III OsU6* (*OsU6a* and *OsU6b*) promoter was used to drive the expression of sgRNA for higher genome editing efficiency. Previous reports in monocots showed that Cas9 expression driven by a strong constitutive promoter maize ubiquitin (*Ubi1*) and the rice *U6* (*OsU6*) promoter for sgRNA expression is relatively efficient in generating targeted effects in the transformed plants (T0) [26, 46, 47].

A pYLCRISPRCas9PUbi-H:OsFAD2 vector with two sgRNA expression cassettes was efficiently assembled in one round of cloning by the Gibson assembly. The integration of the CRISPR/Cas9 cassettes was confirmed by PCR and Sanger sequencing. The vector was tested through the PEG-protoplast-mediated transformation and was then successfully used to create mutations in the stable transgenesis by using a biolistic transformation system. The transgenic lines generated were randomly picked and further genotyped by genomic PCR and followed by direct Sanger sequencing to determine the mutation efficiency. Knocking out the two sgRNAs targeting a single gene caused large deletion of DNA fragments between two target sites and subsequently generated homozygous mutant allele T0 rice calli. This result is also consistent with a previous study which reported the generation of homozygous mutants in the rice T0 [34]. Rice plants with high oleic acid have been developed by other research groups using different approaches, including RNAi [13] and CRISPR/Cas9 single sgRNA gene editing [12]. The oleic acid content was increased to 51.7% as compared to wild type ~ 32% via RNAi technology and increased to approximately 80% via the CRISPR/Cas9 experiment.

Numerous reports of CRISPR/Cas9 targeting of the *FAD2* gene have been published including in *Camelina sativa* [48, 49], soybean (*Glycine max*) [50, 51], *Brassica napus* [52], peanut [53], and tobacco [54]. Results indicated that mutations were obtained at the specific sites in the *FAD2* gene at any location by gene knockout. Moreover, the mutation efficiency mediated by the CRISPR/ Cas9 system varies in different plant species [36, 14]. This finding augurs the finding by Doudna et al. [55], which indicated that the CRISPR/Cas9 genome editing strategy is a way to precisely edit and modify any region of the genome in any species. Later, this technology will be applied to oil palm with the aim to produce high oleic acid content. For this purpose, we aim to knock out the activities of the two key enzymes regulating oil palm fatty acid composition, namely the *FAD2* and palmitoyl-ACP- thioesterase (*PAT*) genes [40–42]. This strategy would greatly reduce the FAD2 and PAT enzyme activities and is expected to increase the oleic acid content to more than 70% with a concomitant decrease of linoleic acid and other long-chain polyunsaturated fatty acids. A high expression of Cas9 and sgRNA driven by plant endogenous promoters is essential for developing the CRISPR/Cas9 system in plants. The *Pol III* promoter (U6) of oil palm will be used to control the expression of sgRNA since the use of endogenous U6P promoter increased the gene-editing efficiency 4–6-fold as demonstrated in soybean [50] and rice [17]. The oil palm constitutive promoters (UEP1, UEP2, TCTP), which were isolated and characterized previously [41], will also be incorporated in the CRISPR/Cas9 vector to control the Cas9 gene. The strategies are expected to increase the gene-editing efficiency of oil palm significantly.

## Conclusion

CRISPR/Cas9 genome editing technology was tested in rice as a model monocot plant for oil palm using a robust CRISPR/Cas9 system utilizing plant codon-optimized *Cas9* gene. Two sgRNAs were designed to knock out the *OsFAD2-1* gene in rice using both PEG-mediated transfection and particle bombardment. Large deletions of approximately 300 bp were detected by PCR and further confirmed by Sanger sequencing in both transformed rice protoplasts and rice calli. A homozygous mutation identified in the T0 generation in rice suggested that the multiplex CRISPR/Cas9 system is highly active in rice, which may be related to the targeting efficiency of different sgRNAs. This study suggested a robust CRISPR/Cas9 vector system could become a powerful tool and can be utilized for efficient multiplex genome editing in future applications. The successful application of genome editing using the CRISPR/Cas9 approach may provide effective solutions for the more complicated crop. This study paves the way for future research in developing oil palm to produce higher oleic acid via Cas9/sgRNA-mediated knockouts of the fatty acid desaturase genes.

## Supplementary Information

**Additional file 1: Supplementary Table 1. List of primers used in this study. Supplementary Fig. 1** Regeneration of rice (*O. sativa* L.) cultivar Nipponbare used for CRISPR/Cas9 sgRNA transformation. Rice seedling growth stages, seed (A & B) germination (C), sprout (D) and seedling (E & F). Calli regenerated from mature seed used for biolistic mediated transformation, before (G) and after (H) bombardment. **Supplementary Fig. 2** Construction of pYLCRISPRCas9PubiH-OsFAD. (a) pYLCRISPR/Cas9PubiH vector backbone digested with *Bsa*I for insertion of sgRNAs. (b) First-round amplification of each sgRNA expression cassette, bands at ~600 bp (Lane 1: pYLsgRNAOsU6a) and ~500 bp (Lane 2: pYLsgRNAOsU6b) were amplified using the U-F/sgRNA reverse primers. (c) The bands at ~150 bp (Lanes 1 and 2) were amplified using the sgRNA forward /GR-R primers. PCR amplification of two sgRNAs expression cassettes, (d) U6aOsFAD2T1 and (e) U6bOsFAD2T2. (f) Verification of the CRISPR/Cas9 positive clones with *Asc*1 digestion. Lane M in (a), (b), (d), (e) and (f ) is Trans2K® Plus II DNA Marker (TransGen Biotech, China). Lane M in (c) is 100 bp Plus DNA ladder (TransGen Biotech, China). (e) The overall cloning strategy for construction of pYLCRISPRCas9Pubi-H:OsFAD2 using overlapping PCR to combine two sgRNAs expression cassettes and then cloned into pYLCRISPR/Cas9PubiH vector via gibson assembly cloning method. LB: left border, *HPT*: gene for hygromycin phosphotransferase, 2xP*35S*: Double cauliflower mosaic virus *35S* promoter, P*ubi*: maize ubiquitin *Ubi1* promoter, *Cas9*p: CRISPR associated protein 9, *U6a*: rice (*O. sativa*) *U6a* (*OsU6a*) promoter, sgRNA1: OsFAD2-T1, *U6b*: rice (*O. sativa*) *U6b* (*OsU6b*) promoter, sgRNA2: OsFAD2-T2, RB: right border). **Supplementary Fig. 3** Recombinant screening analysis of pYLCRISPRCas9PubiH-OsFAD vector. (A) Gel analysis of recombinants via colony PCR using OsFAD2U6bT2R and OsFAD2U6aT1F primers. Lanes 1 to 11 are colonies purified and cloned. A red arrow with a size of 500 bp indicates PCR products. Lane M is Trans2K® Plus II DNA Marker (TransGen, Beijing, China). (B) The output from Sanger sequencing analysis. Underlined sequences consist of OsFAD2-T1 and OsFAD2-T2 sgRNAs. Orange and red letters indicated the *OsU6a* and *OsU6b* promoters, respectively. **Supplementary Fig. 4** Molecular analysis of T0 transgenic rice calli. (A) The PCR products of 767 bp and 474 bp amplified using FAD2-F2 and FAD2-R primers. Lane M is Trans2K® Plus II DNA Marker (TransGen Biotech, China). (B) Sequence alignment for detection of mutations at target sites of OsFAD2-T1 and OsFAD2-T2. Sequence analysis of the edited line indicated in the light red box and dotted lines (….) represents deletions. The red and orange boxes are the target sequences, and the PAM sequence is shown in blue underlined nucleotides. (C) A schematic representation of the residue of target 1 (OsFAD2-T1) in the red box and the residue of target 2 (OsFAD2-T2) in the orange box. **Supplementary Fig. 5** (A) Sequencing result decoded homozygous mutations in both alleles. (B) Representative chromatograms of PCR product from homozygous mutant alleles with expected (i) 302 nucleotides deletion for amplicon 474 bp and (ii) 1 nucleotide insertion for amplicon 776 bp in the T0 rice.

## Data Availability

The authors declare that all data generated or analysed in this study are included in the article.

## Abbreviations

Cas9: CRISPR-associated protein 9
CRISPR: Clustered regularly interspaced short palindromic repeats
FAD2: Oleoyl-CoA desaturase
OsFAD2: *Oryza sativa* FAD2
PAM: Protospacer adjacent motif
PEG: Polyethylene glycol
sgRNA: Single-guide RNA
