# Supplementary material for: Multiplex CRISPR/Cas9-mediated genome editing of the FAD2 gene in rice: a model genome editing system for oil palm
Source: J Genet Eng Biotechnol. 2021 Jun 11;19:86. doi: 10.1186/s43141-021-00185-4 (PMC8196110; doi:10.1186/s43141-021-00185-4)
Supplement: Supplementary file 1 — Additional file 1: Supplementary Table 1. List of primers used in this study. Supplementary Fig. 1 Regeneration of rice (O. sativa L.) cultivar Nipponbare used for CRISPR/Cas9 sgRNA transformation. Rice seedling growth stages, seed (A & B) germination (C), sprout (D) and seedling (E & F). Calli regenerated from mature seed used for biolistic mediated transformation, before (G) and after (H) bombardment. Supplementary Fig. 2 Construction of pYLCRISPRCas9PubiH-OsFAD. (a) pYLCRISPR/Cas9PubiH vector backbone digested with BsaI for insertion of sgRNAs. (b) First-round amplification of each sgRNA expression cassette, bands at ~600 bp (Lane 1: pYLsgRNAOsU6a) and ~500 bp (Lane 2: pYLsgRNAOsU6b) were amplified using the U-F/sgRNA reverse primers. (c) The bands at ~150 bp (Lanes 1 and 2) were amplified using the sgRNA forward /GR-R primers. PCR amplification of two sgRNAs expression cassettes, (d) U6aOsFAD2T1 and (e) U6bOsFAD2T2. (f) Verification of the CRISPR/Cas9 positive clones with Asc1 digestion. Lane M in (a), (b), (d), (e) and (f ) is Trans2K® Plus II DNA Marker (TransGen Biotech, China). Lane M in (c) is 100 bp Plus DNA ladder (TransGen Biotech, China). (e) The overall cloning strategy for construction of pYLCRISPRCas9Pubi-H:OsFAD2 using overlapping PCR to combine two sgRNAs expression cassettes and then cloned into pYLCRISPR/Cas9PubiH vector via gibson assembly cloning method. LB: left border, HPT: gene for hygromycin phosphotransferase, 2xP35S: Double cauliflower mosaic virus 35S promoter, Pubi: maize ubiquitin Ubi1 promoter, Cas9p: CRISPR associated protein 9, U6a: rice (O. sativa) U6a (OsU6a) promoter, sgRNA1: OsFAD2-T1, U6b: rice (O. sativa) U6b (OsU6b) promoter, sgRNA2: OsFAD2-T2, RB: right border). Supplementary Fig. 3 Recombinant screening analysis of pYLCRISPRCas9PubiH-OsFAD vector. (A) Gel analysis of recombinants via colony PCR using OsFAD2U6bT2R and OsFAD2U6aT1F primers. Lanes 1 to 11 are colonies purified and cloned. A red arrow with a size of 500 bp indi [file 43141_2021_185_MOESM1_ESM.docx]

**Supplementary Table 1. List of primers used in this study**

| **Usage** | **Primer name** | | **Primer sequence (5’-3’)** |
| --- | --- | --- | --- |
| Primers for amplification of target region | OsFAD2#220F | | CTCTACTTCGCGCTGGTCAT |
|  | OsFAD2#1136R | | TTGTTGTCCTCAGGCTCGAC |
| Primers for construction of sgRNA expression cassettes | OsFAD2U6aT1F | | gccgTACGTGTACCACAACCCGAT |
|  | OsFAD2U6aT1R | | aaacATCGGGTTGTGGTACACGTA |
|  | OsFAD2U6bT2F | | gttgCTACCTGCAGCACACCCACC |
|  | OsFAD2U6bT2R | | aaacGGTGGGTGTGCTGCAGGTAG |
| Forward primers to generate DNA template for *in vitro* transcription | OsFAD2T1Forward | | CCTCTAATACGACTCACTATAGGTACGTG  TACCACAACCCGATGTTTAAGAGCTATGC |
|  | OsFAD2T2Forward | | CCTCTAATACGACTCACTATAGGCTACCT  GCAGCACACCCACCGTTTAAGAGCTATGC |
| Primers used for Gibson Assembly of sgRNA expression cassettes | 1^st^ PCR | U-F | CTCCGTTTTACCTGTGGAATCG |
|  |  | gR-R | CGGAGGAAAATTCCATCCAC |
|  | 2^nd^ PCR | U-GAL | ACCGGTAAGGCGCGCCGTAGTGCTC  GACTAGTATGGAATCGGCAGCAAAGG |
|  |  | Pgs-GA2 | CAGGGAGCGGATAACAATTTCA  CACAGGCACATCCACTCCAAGCTCTTG |
|  |  | U-GA2 | GTGCCTGTGTGAAATTGTTATCCGCT  CCCTGGAATCGGCAGCAAAGG |
|  |  | Pgs-GAR | TAGCTCGAGAGGCGCGCCAATGATACCGAC  GCGTATCCATCCACTCCAAGCTCTTG |
| Primers for Sanger sequencing | SP-L1 | | GCGGTGTCATCTATGTTACTAG |
|  | SP-R | | CGAAGTTATTGCATCTATGTCG |
| Primers for analysis on target gene mutations | FAD-F | | CACCACTCCAACACCGGGTCGCTG |
|  | FAD-R | | TGTGGAACACCTTGTTGAGGATGCCGTAG |
|  | FAD2-F-2 | | CTTCTCCTACGTGGTCCATGACCTCGTGA |

**
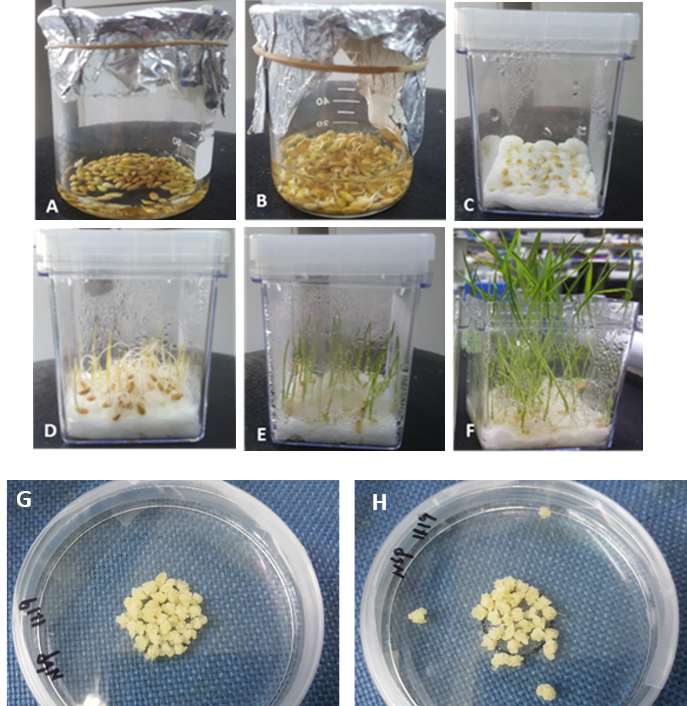
**

**Supplementary Fig. 1** Regeneration of rice (*O. sativa* L.) cultivar Nipponbare used for CRISPR/Cas9 sgRNA transformation. Rice seedling growth stages, seed (A & B) germination (C), sprout (D) and seedling (E & F). Calli regenerated from mature seed used for biolistic mediated transformation, before (G) and after (H) bombardment.


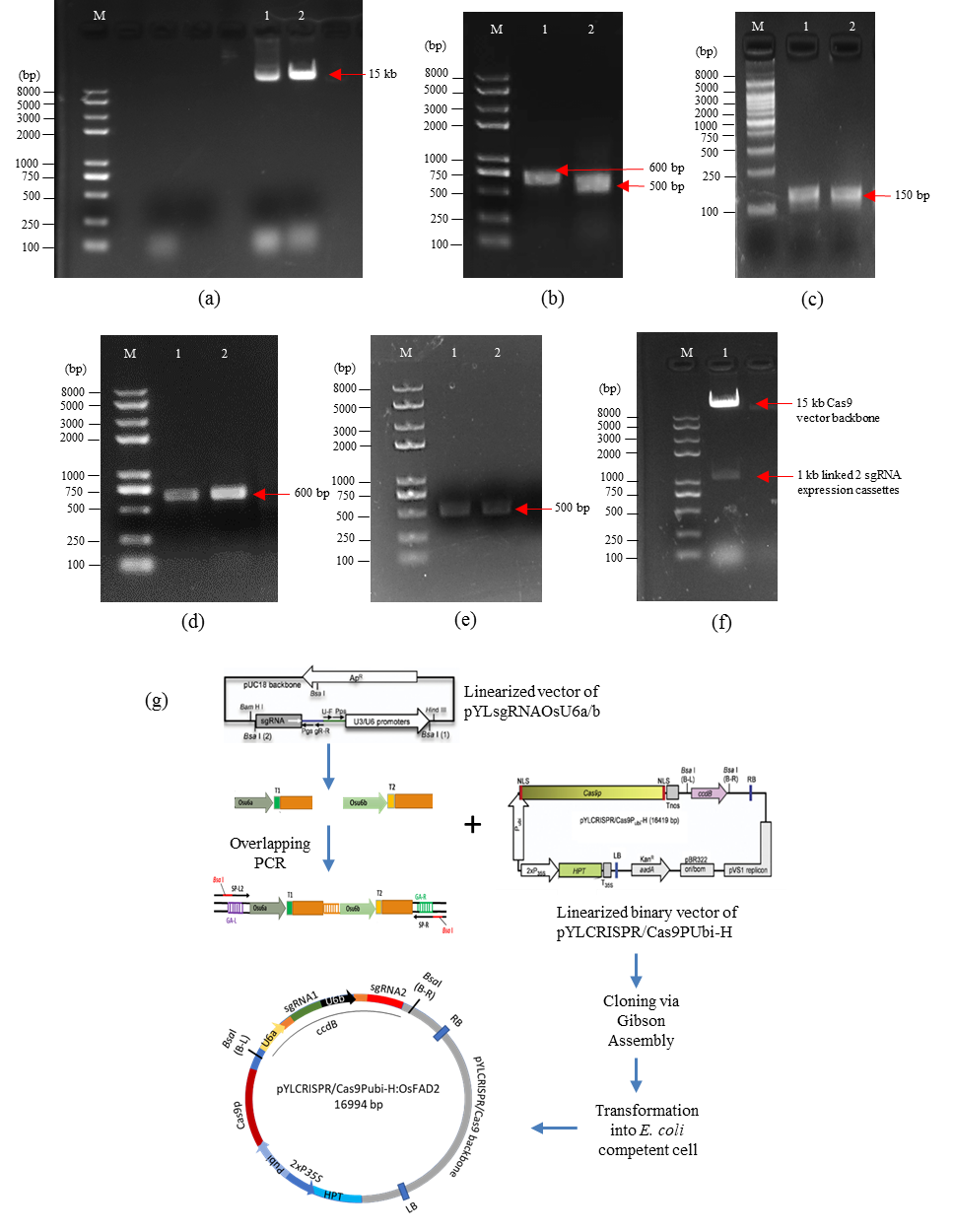


**Supplementary Fig. 2** Construction of pYLCRISPRCas9PubiH-OsFAD. (a) pYLCRISPR/Cas9PubiH vector backbone digested with *Bsa*I for insertion of sgRNAs. (b) First-round amplification of each sgRNA expression cassette, bands at ~600 bp (Lane 1: pYLsgRNAOsU6a) and ~500 bp (Lane 2: pYLsgRNAOsU6b) were amplified using the U-F/sgRNA reverse primers. (c) The bands at ~150 bp (Lanes 1 and 2) were amplified using the sgRNA forward /GR-R primers. PCR amplification of two sgRNAs expression cassettes, (d) U6aOsFAD2T1 and (e) U6bOsFAD2T2. (f) Verification of the CRISPR/Cas9 positive clones with *Asc*1 digestion. Lane M in (a), (b), (d), (e) and (f ) is Trans2K^®^ Plus II DNA Marker (TransGen Biotech, China). Lane M in (c) is 100 bp Plus DNA ladder (TransGen Biotech, China). (e) The overall cloning strategy for construction of pYLCRISPRCas9Pubi-H:OsFAD2 using overlapping PCR to combine two sgRNAs expression cassettes and then cloned into pYLCRISPR/Cas9PubiH vector via gibson assembly cloning method. LB: left border, *HPT*: gene for hygromycin phosphotransferase, 2xP*35S*: Double cauliflower mosaic virus *35S* promoter, P*ubi*: maize ubiquitin *Ubi1* promoter, *Cas9*p: CRISPR associated protein 9, *U6a*: rice (*O. sativa*) *U6a* (*OsU6a*) promoter, sgRNA1: OsFAD2-T1, *U6b*: rice (*O. sativa*) *U6b* (*OsU6b*) promoter, sgRNA2: OsFAD2-T2, RB: right border).


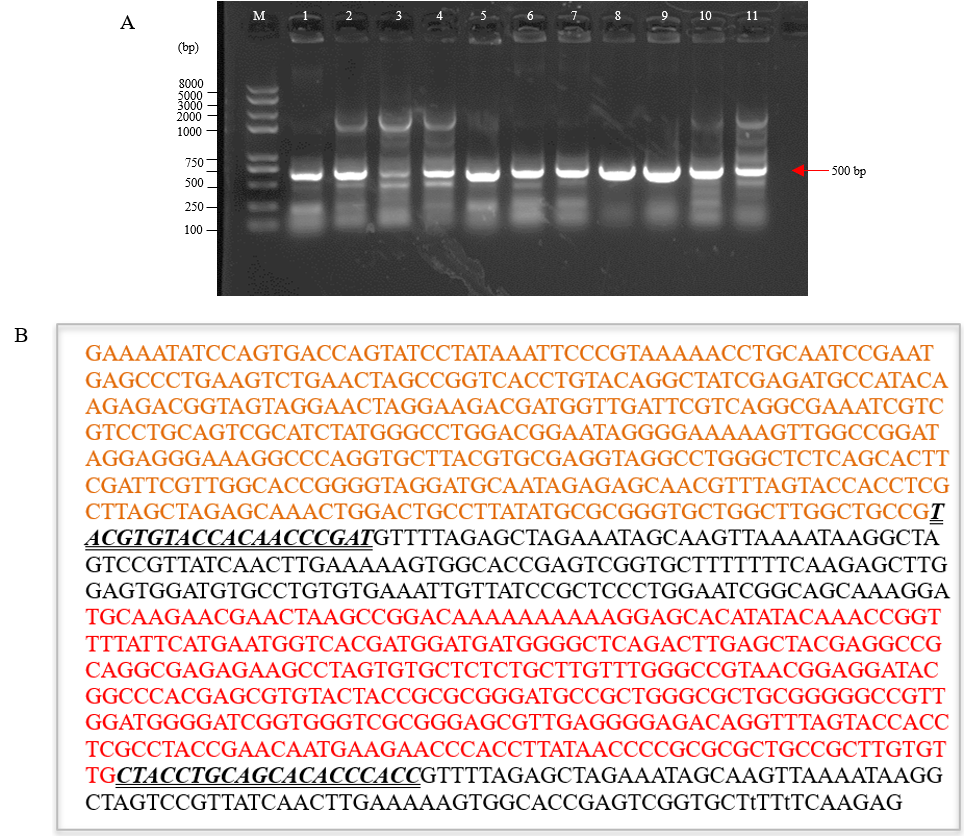


+

**Supplementary Fig. 3** Recombinant screening analysis of pYLCRISPRCas9PubiH-OsFAD vector. (A) Gel analysis of recombinants via colony PCR using OsFAD2U6bT2R and OsFAD2U6aT1F primers. Lanes 1 to 11 are colonies purified and cloned. A red arrow with a size of 500 bp indicates PCR products. Lane M is Trans2K^®^ Plus II DNA Marker (TransGen, Beijing, China). (B) The output from Sanger sequencing analysis. Underlined sequences consist of OsFAD2-T1 and OsFAD2-T2 sgRNAs. Orange and red letters indicated the *OsU6a* and *OsU6b* promoters, respectively.


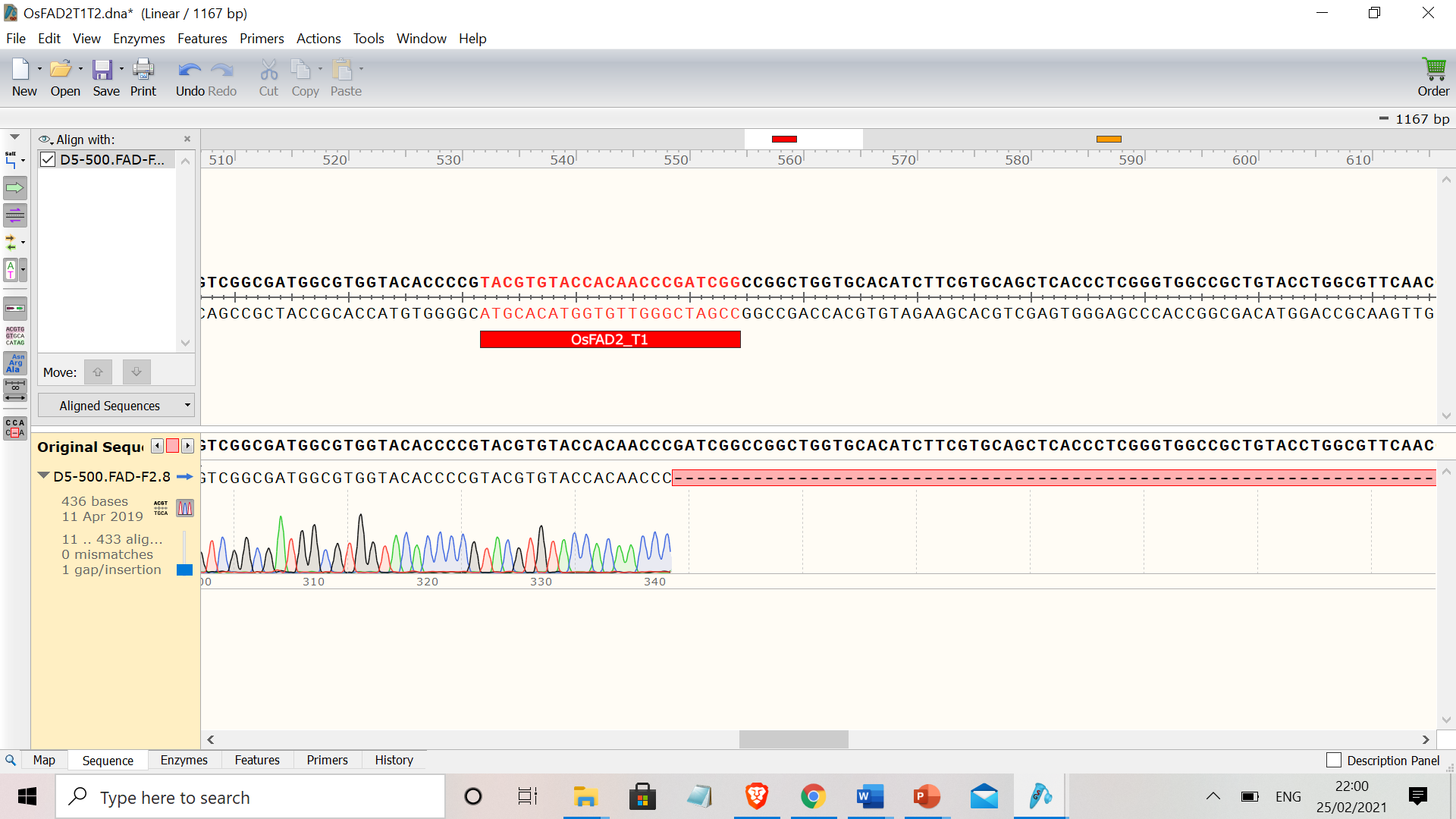

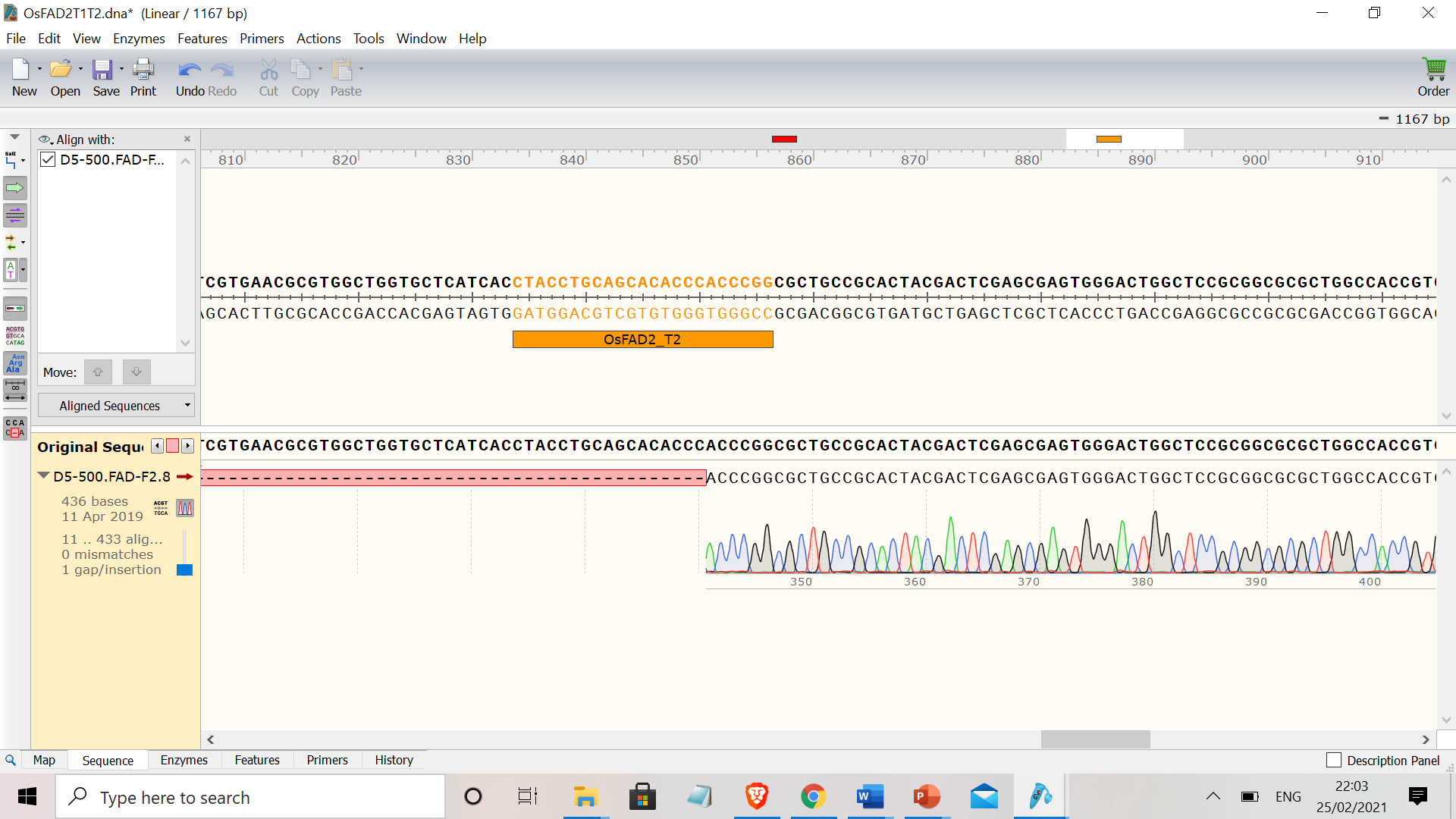


**302 bp**


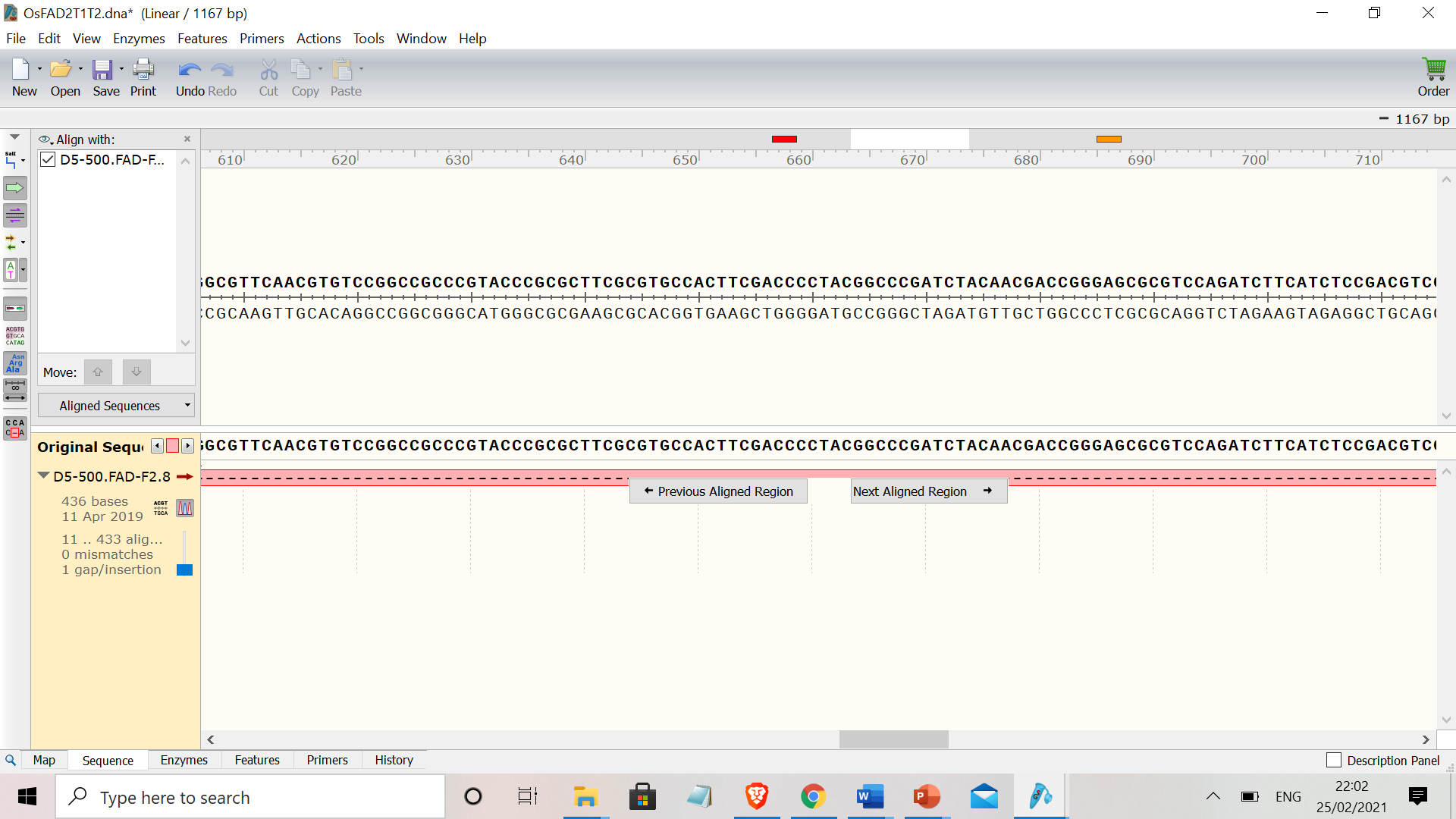


**WT**

**WT**

**5’**

**3’**

**PAM**

**PAM**

**sgRNA1**

**sgRNA2**

**OsFAD2_D5**

B


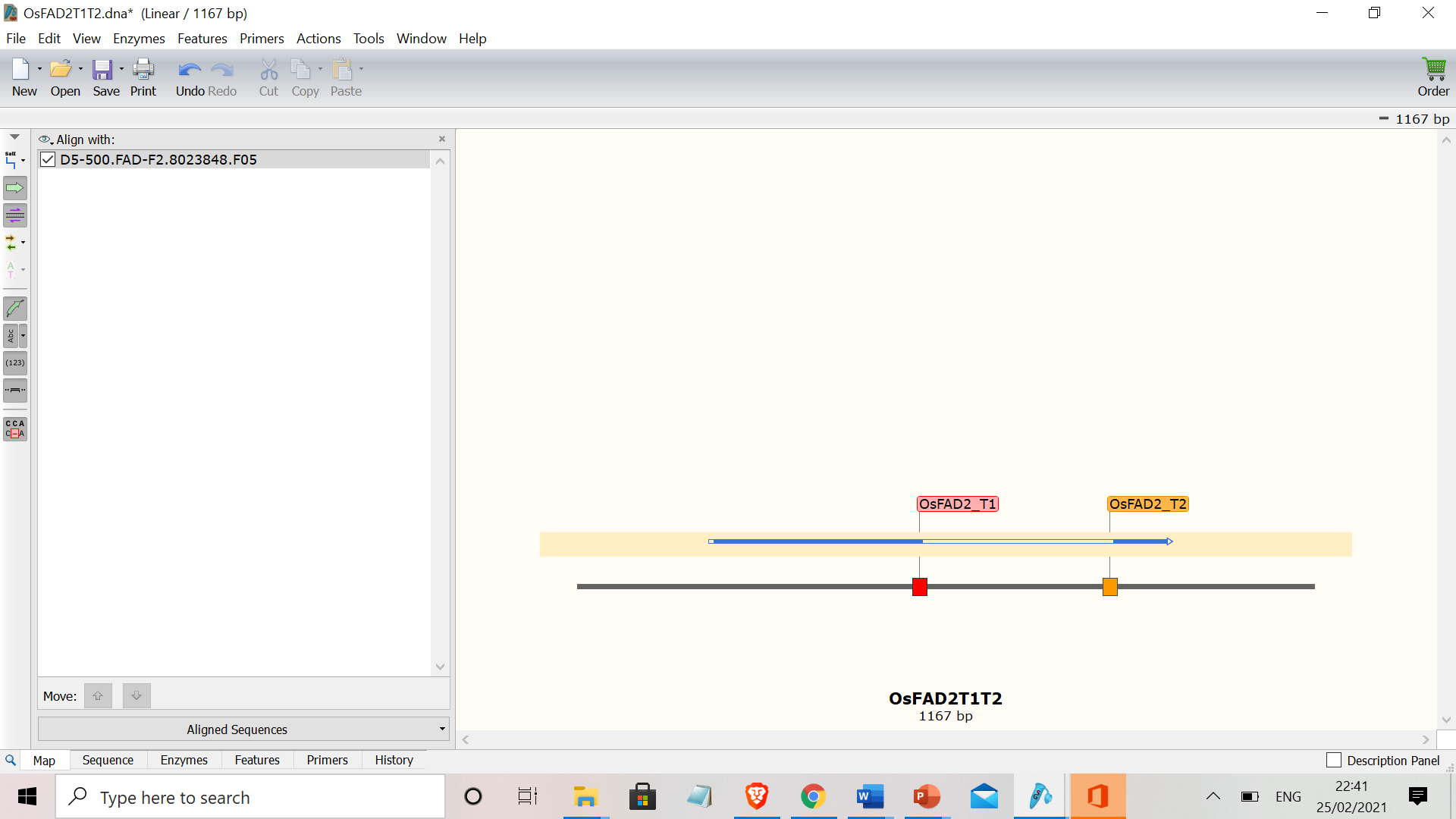


**Residue of Target 2**

**Residue of Target 1**

C


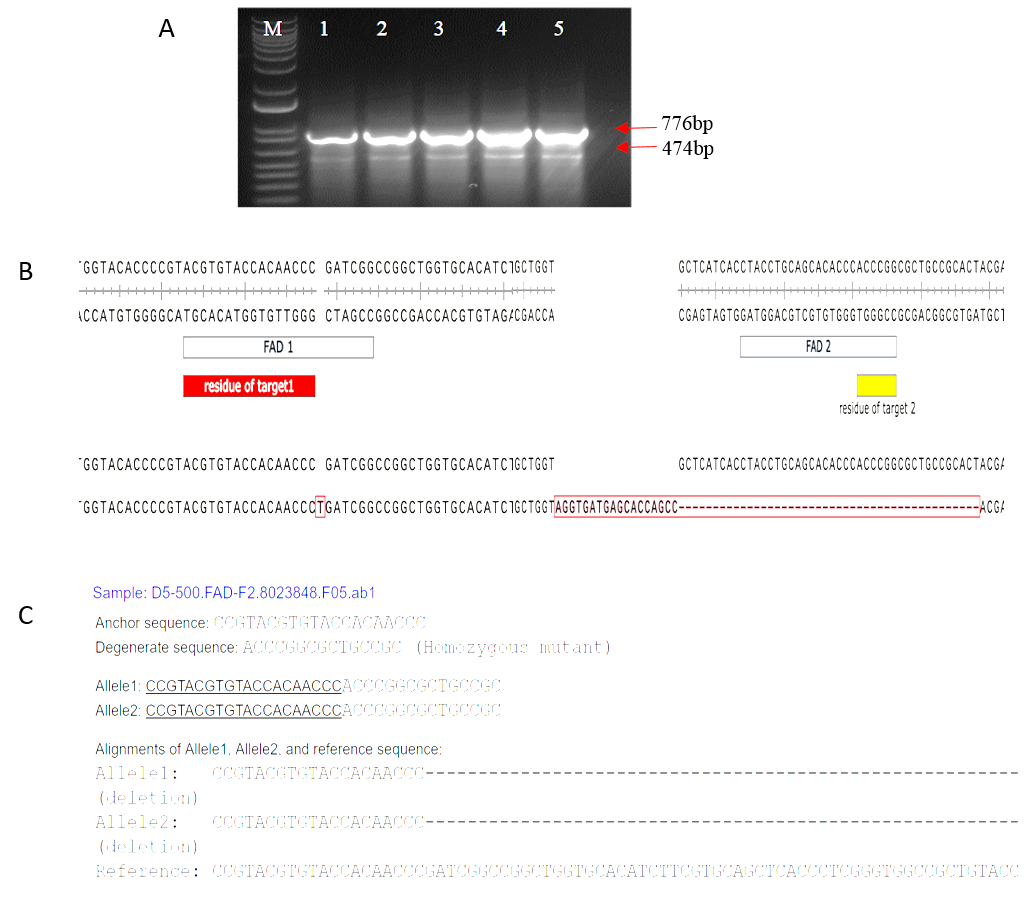


A

100

200

1200

900

800

(bp)

1000

300

400

500

600

700

1500

**Supplementary Fig. 4** Molecular analysis of T0 transgenic rice calli. (A) The PCR products of 767 bp and 474 bp amplified using FAD2-F2 and FAD2-R primers. Lane M is Trans2K^®^ Plus II DNA Marker (TransGen Biotech, China). (B) Sequence alignment for detection of mutations at target sites of OsFAD2-T1 and OsFAD2-T2. Sequence analysis of the edited line indicated in the light red box and dotted lines (….) represents deletions. The red and orange boxes are the target sequences, and the PAM sequence is shown in blue underlined nucleotides. (C) A schematic representation of the residue of target 1 (OsFAD2-T1) in the red box and the residue of target 2 (OsFAD2-T2) in the orange box.


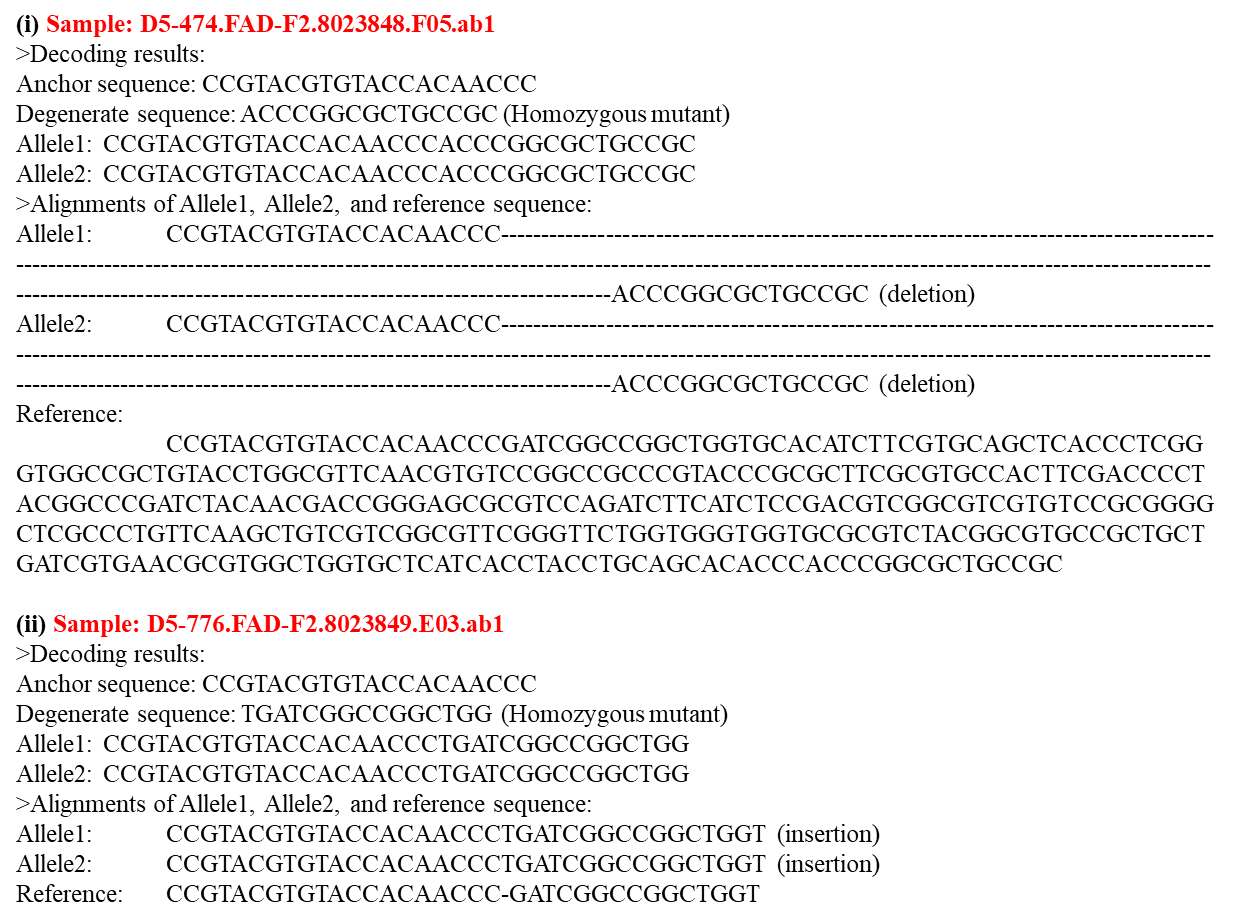


A


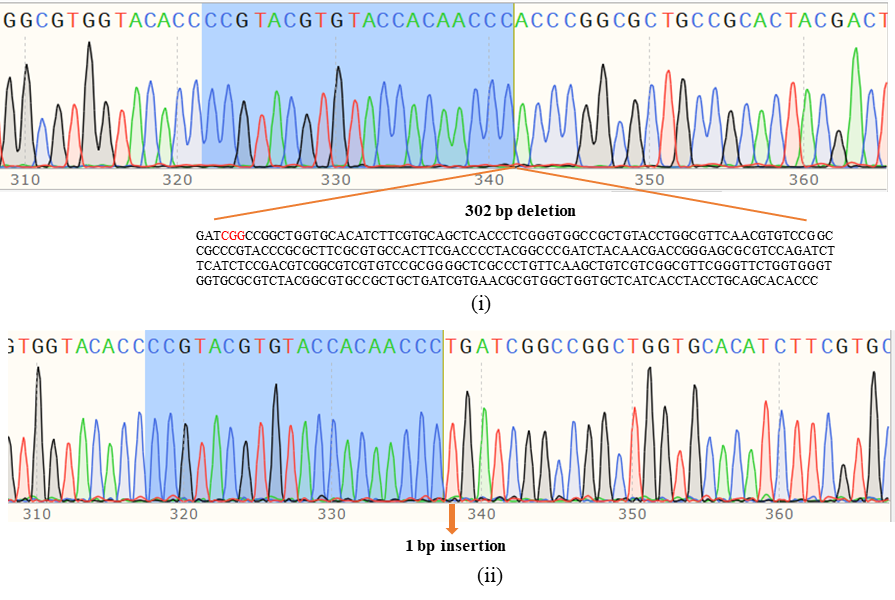


B

**Supplementary Fig. 5** (A) Sequencing result decoded homozygous mutations in both alleles. (B) Representative chromatograms of PCR product from homozygous mutant alleles with expected (i) 302 nucleotides deletion for amplicon 474 bp and (ii) 1 nucleotide insertion for amplicon 776 bp in the T0 rice.
